# Optimization of Fuzzy Control Parameters for Wind Farms and Battery Energy Storage Systems Based on an Enhanced Artificial Bee Colony Algorithm under Multi-Source Sensor Data

**DOI:** 10.3390/s24165115

**Published:** 2024-08-07

**Authors:** Zejian Liu, Ping Yang, Peng Zhang, Xu Lin, Jiaxi Wei, Ning Li

**Affiliations:** 1 Key Laboratory of Clean Energy Technology of Guangdong Province, School of Electric Power Engineering, South China University of Technology, Guangzhou 510640, China; liu.zejian@hgnyjs.com (Z.L.); eppyang@scut.edu.cn (P.Y.); zp1979377074@sina.com (P.Z.); 2 Shenzhen Huagong Energy Technology Co., Ltd., Shenzhen 518066, China; 3 Power Dispatch Control Center, Guangdong Power Grid Corporation, Guangzhou 510699, China; 18814091950@163.com; 4 School of Electrical Engineering, Xi’an University of Technology, Xi’an 710048, China; 17791029549@163.com

**Keywords:** DFIG, primary frequency control, fuzzy membership function, multi-source sensor data, ABC algorithm, Gaussian walk mechanism

## Abstract

With the rapid development of sensors and other devices, precise control for the generation of new energy, especially in the context of highly stochastic wind power generation, has been strongly supported. However, large-scale wind farm grid connection can cause the power system to enter a low inertia state, leading to frequency instability. Battery energy storage systems (BESSs) have the advantages of a fast response speed and high flexibility, and can be applied to wind farm systems to improve the frequency fluctuation problem in the process of grid connection. To address the frequency fluctuation problem caused by the parameter error of the fuzzy membership function in the fuzzy control of a doubly fed induction generator (DFIG) and a BESS, this paper proposes an improved Artificial Bee Colony (ABC) algorithm based on multi-source sensor data for optimizing the fuzzy controller to improve the frequency control ability of BESSs and DFIGs. A Gaussian wandering mechanism was introduced to improve the ABC algorithm and enhance the convergence speed of the algorithm, and the improved ABC algorithm was optimized for the selection of fuzzy control affiliation function parameters to improve the frequency response performance. The effectiveness of the proposed control strategy was verified on the MATLAB/Simulink simulation platform. After optimization using the proposed control strategy, the oscillation amplitude was reduced by 0.15 Hz, the precision was increased by 40%, and the steady-state frequency deviation was reduced by 26%. The results show that the method proposed in this paper provides a great improvement in the frequency stability of coordinated systems of wind farms and BESSs.

## 1. Introduction

The development of renewable energy is of great significance in addressing global energy crises and climate change. With advancements in measuring, and responsive devices such as sensors, the renewable energy generation represented by wind power has seen significant improvements in electricity generation efficiency, response speed, and control precision. Because wind power has the problems of randomness, intermittency, and uncontrollability [1,2], large-scale grid integration will have an impact on the grid frequency, which, in turn, affects the stable operation of the whole grid [3]. However, the advantages of energy storage technology, such as a fast response time and high flexibility, have a good effect on improving the frequency fluctuation in the process of grid connection [4,5,6,7].

Although energy storage optimizes the problem of frequency fluctuations, it still has many shortcomings. At present, the research hotspots at home and abroad regarding the primary frequency modulation of coordinated systems of wind farms and BESSs are divided into traditional control and algorithmic control according to the different categories of technology used. Traditional control methods refer to the control system only through various types of converters and pitch angle control, active power control, virtual inertia control, and other typical control methods for primary FM control. In [8], the authors proposed a coordinated control strategy between a small battery energy storage system (BESS) and PMSG-WTG based on the torque limit curve inertia control method. This method eliminates the problem of frequency reduction in the process of rotor speed recovery and maximizes the inertia response under the condition of large-scale variable wind speed. In [9], a coordinated control strategy was proposed for short-term power spikes such as virtual inertia control for wind turbines and steady-state power for energy storage, fully utilizing rotor inertia to reduce the required energy storage capacity and improve economic efficiency. In [10], a strategy for wind farms and BESSs based on standby demand and state reconstruction was proposed, in which the state of charge (SOC) is partitioned. This strategy avoids the high-power charging and discharging of the energy storage battery outside the normal state, which is conducive to maintaining the SOC of the battery and its state of health, thus delaying the aging of the battery and improving its operational life. In [11], a coordinated control strategy based on the voltage source converter using a BESS to provide frequency support was proposed. The voltage of the DC circuit is adjusted through the high-voltage direct current connection to support the frequency modulation. In [12], a new hybrid operation strategy of a wind energy conversion system (WECS) and a BESS was proposed. A machine-side converter (MSC) and a grid-side converter (GSC) were designed to achieve maximum power extraction. The output power command of the BESS is determined based on the SOC, frequency deviation, and load variation.

Traditional control methods increase the complexity and economic cost of the whole system [13,14]. With the continuous development of artificial intelligence, various types of algorithms such as neural networks, deep learning, particle swarm optimization, and other algorithms have also launched relevant applications in the field of the coordinated frequency of wind farms and BESSs [15,16,17,18,19,20]. Optimizing control by adding algorithms can greatly simplify the control process, improve system response, reduce system energy consumption, and provide better stability. In [21], an adaptive control strategy based on MOPSO was proposed. Based on the Model Predictive Control (MPC) optimization technology, the gain of the system controller is adjusted online to respond to the frequency fluctuation caused by load fluctuation. In [22], the authors proposed an energy management strategy for a hybrid energy storage system of remote area power supply (RAPS) system with wind farm control. The depth of discharge (DOD) and ripple content of the battery current are reduced through an energy management algorithm (EMA).

In addition, fuzzy control is more suitable for the coordinated system of wind farms and BESSs due to its mature technology, simple control, and other advantages [23,24]. In [25], a new control strategy to compensate for the inertia of wind farms was proposed, and fuzzy control is applied to realize the recovery of rotor speed. In [26], a fuzzy-logic-based frequency controller (FFC) for wind farms was proposed to enhance BESSs, ensuring the optimal utilization of energy in wind farms and storage units by eliminating the flexible offloading of wind energy and minimizing the storage capacity required for a given frequency support level. In [27], a control strategy for the coordinated system of a wind farm and a BESS to participate in the primary frequency modulation of the power grid was proposed. Fuzzy logic control (FLC) is added to the coordinated frequency modulation system of a wind farm and a BESS. The fuzzy logic controller is designed with wind speed, the frequency difference rate of change, and the frequency deviation as fuzzy inputs, and the dynamic frequency modulation under different SOC states is realized. In [28], a control scheme based on the Fuzzy Gain Scheduling PI (FGS-PI) model, and the improved Gray Wolf Optimization (GWO) algorithm is proposed. An adaptive fuzzy PI controller is used to manage the optimal power of supercapacitors and batteries in a PV generation system. However, the existing membership functions of the primary frequency modulation of wind farms and BESSs using fuzzy control are based on the experience of experts and experiments. A large number of tests are required to adjust the parameters related to the membership function, which can easily cause a waste of resources.

In order to solve the above problems, this paper proposes an optimization of fuzzy control parameters for wind farms and BESSs based on an enhanced ABC algorithm with multi-source sensor data. Firstly, a primary frequency modulation strategy based on fuzzy control was designed to effectively solve the frequency modulation problem of wind farms under frequency fluctuation. Secondly, the Lévy flight and Gaussian walk mechanism are introduced into the ABC algorithm to optimize the algorithm in order to avoid falling into the local solution and improve the speed of the algorithm. Finally, for the key parameters of the fuzzy control membership function, the improved ABC algorithm is used to optimize the parameters to obtain the most suitable membership function.

The contributions of this paper are as follows:

(1) This paper proposes a coordinated and unified control model for wind farms and BESSs. The fuzzy control dynamic adjustment system is applied to adjust the primary frequency modulation coefficient.
(2) The ABC algorithm is used to design the optimal fuzzy logic controller to enhance the frequency control performance of the energy storage module.
(3) The ABC algorithm is improved to enhance the accuracy and speed of the algorithm.

The rest of this paper is structured as follows. Section 2 establishes a hybrid control model consisting of a doubly fed wind farm and a BESS module. Section 3 describes the specific logical steps of the improved ABC algorithm to obtain the optimized fuzzy logic control function. In Section 4, the established model is simulated and the results are discussed, and the effectiveness of the proposed control strategy is verified.

## 2. Coordinated Primary Frequency Modulation Control Strategy of a Wind Farm and a BESS

### 2.1. Frequency Domain Analysis of a Coordinated Frequency System of a DFIG and a BESS

**Figure 1:**
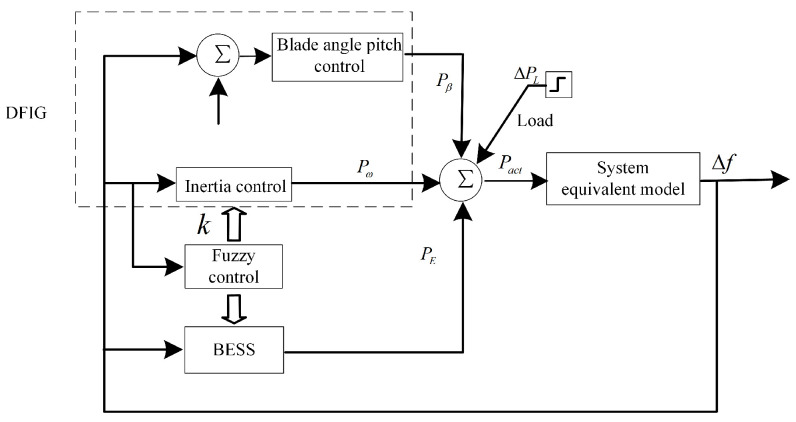
Combined control strategy of the DFIG and the BESS.

The control schemes used in this paper for DFIGs include inertial control, a blade angle pitch controller, and a fuzzy logic controller to coordinate the output of active power. Inertial control here refers to the control of rotor speed changes through the rotor side converter, with the short-term release or absorption of stored kinetic energy in the wind turbine, in order to quickly respond to system frequency changes. Figure 1 shows the schematic diagram of the coordinated control strategy of the DFIG and the BESS.

The mismatch of active power in the system is a major cause of generator downtime, line tripping, and load dropping. Inertia control ensures that the generator speed is synchronized with the grid frequency. Blade angle pitch control ensures maximum power generation efficiency under different wind speed conditions. By combining them, the DFIG has the inertia response and the ability for primary frequency modulation similar to the conventional generator set. The frequency-domain transfer function of the DFIG is (1).

(1) $$\begin{array}{c} G_{W}\left(s\right)=\frac{\Delta P_{\omega }+\Delta P_{\beta }}{\Delta f}=\left(-\frac{k_{\mathrm{df}}s}{1+T_{\omega }s}\right)+\left(-\frac{k_{\mathrm{pf}}}{1+T_{\beta }s}\right)\\ =-\frac{\left(k_{\mathrm{df}}T_{\beta }\right)s^{2}+\left(k_{\mathrm{df}}+k_{\mathrm{pf}}T_{\omega }\right)s+k_{\mathrm{pf}}}{T_{o}T_{\beta }s^{2}+\left(T_{\omega }+T_{\beta }\right)s+1} \end{array}$$

where $\Delta P_{\omega }$ and $\Delta P_{\beta }$ are the rotor inertia power and blade angle pitch control power and $k_{\mathrm{df}}$ and $k_{\mathrm{pf}}$ are the inertia response coefficient and the primary frequency modulation coefficient, respectively. $T_{\omega }$ and $T_{\beta }$ are the rotor inertia response time constant and the blade angle pitch control response time constant, respectively.

With the gradual decrease in the frequency change rate, the active support role of DFIG on the system is weakened. At this time, the output characteristics of the energy storage just make up for the lack of DFIG output decline. During the frequency homeostasis recovery phase, the DFIG exits frequency modulation. Energy storage will continue to provide power support and speed up the recovery of system frequencies. The frequency-domain transfer function of the energy storage system is (2).

(2) $$G_{E}\left(s\right)=-\frac{k_{\mathrm{df}}s+k_{\mathrm{pf}}}{T_{E}s+1}$$

where the inertia response coefficient $k_{\mathrm{df}}$ and primary frequency modulation coefficient $k_{\mathrm{pf}}$ of the energy storage system are the same as those of the DFIG. $T_{E}$ is the energy storage response time constant.

The median value theorem is (3).

(3) $$\begin{array}{c} \Delta f=\lim_{s\to 0}s\Delta f\left(s\right)=\lim_{s\to 0}s\cdot G_{s}\left(s\right)\cdot \frac{\Delta P_{L}}{s} \end{array}$$

From (3), it is concluded that the frequency deviation of coordinated primary frequency modulation of the DFIG and BESS is (4).

(4) $$\Delta f=\frac{R}{(1-p+pk_{\mathrm{pf}}R)+DR}\Delta P_{L}$$

where *D* is the damping characteristic, *R* is the sagging characteristic, $\Delta P_{L}$ is the size of the perturbation, and *p* is the penetration rate of wind power.

### 2.2. Designed Fuzzy Controller for a Coordinated System of a Wind Farm and a BESS

Fuzzy control does not depend on specific mathematical models to control with good nonlinear effects. In this paper, fuzzy control is added on the basis of a traditional wind farm and a BESS frequency modulation PI regulation system. It dynamically adjusts the primary frequency modulation coefficient of the wind farm and the BESS.

**Figure 2:**
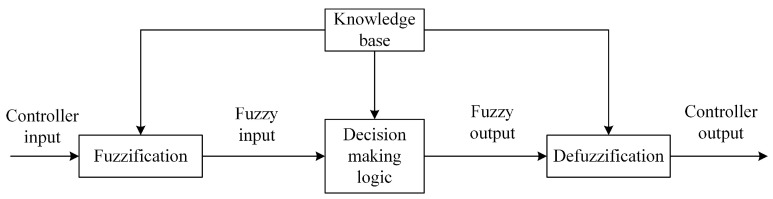
Logic block diagram of fuzzy control.

The basic structure of the fuzzy control system is fuzziness, a knowledge base, a decision-making logic, and defuzzing. Figure 2 is the logic block diagram of fuzzy control, which shows the relationship between the modules of fuzzy control. Fuzzification transforms precise input data into appropriate linguistic values or identifiers of fuzzy sets. The decision-making logic is the core of a fuzzy control system. At this stage, fuzzy relation equations are solved based on fuzzy inputs and fuzzy control rules to obtain fuzzy outputs. Defuzzification primarily converts the inferred fuzzy control quantity into precise control outputs. Initially, fuzzy outputs are defuzzified using methods such as centroids, the maximum membership principle, and the area method. Subsequently, the defuzzified results generate corresponding control signals for actual system control.

The fuzzy control rules are as follows (5):

(5) $$\begin{array}{c} \mu_{R_{i}}\overset{\Delta }{=}\mu_{\left(A_{i}\mathrm{and}B_{i}\to C_{i}\right)}\left(u,v,w\right)\\ =\left[\mu_{A_{i}}\left(u\right)\mathrm{and}\mu_{B_{i}}\left(v\right)\right]\to \mu_{C_{i}}\left(w\right) \end{array}$$

where $A_{i}$ and $B_{i}$ are fuzzy sets that exist in U×V, $R_{i}\overset{\Delta }{=}\left(A_{i}\mathrm{and}B_{i}\right)\to C_{i}$ is the fuzzy relationship in U×V×W, and → is the fuzzy implicit function.

**Figure 3:**
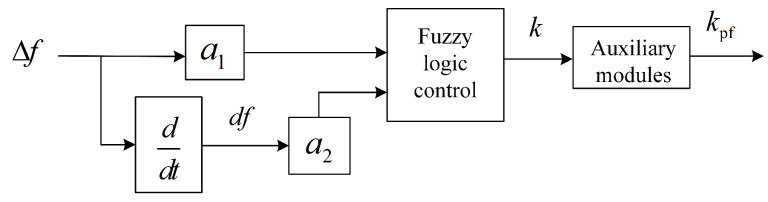
Block diagram of fuzzy control.

Figure 3 shows a two-input, single-output fuzzy control established in this paper. The inputs of the fuzzy membership function are the frequency deviation Δf and the frequency rate of change df/dt, and the output is *k*. Δf represents the amount of frequency change, and df/dt represents the speed of frequency change. Both serve as inputs for fuzzy control and are indispensable. We adjust the primary frequency modulation coefficient $k_{\mathrm{pf}}$ in real time according to frequency fluctuations. The set of Δf and df/dt is $a_{1}\Delta f$ and $a_{2}df/dt$. It is the input into the unit of the membership function. The membership function is (6).

(6) k=γ+ϑΔf+φdf

where

(7) $$\begin{array}{c} \gamma =k_{ij}-\frac{k_{(i+1)j}-k_{ij}}{a_{1}\left(\Delta f_{\left(i+1\right)}-\Delta f_{i}\right)}\Delta f_{i}+\\ \frac{k_{i(j+1)}-k_{ij}}{a_{2}\left(df_{\left(j+1\right)}-df_{j}\right)}df_{j}\\ \vartheta =\frac{k_{(i+1)j}-k_{ij}}{a_{1}\left(\Delta f_{\left(i+1\right)}-\Delta f_{i}\right)}\\ \varphi =\frac{k_{i(j+1)}-k_{ij}}{a_{2}\left(df_{\left(j+1\right)}-df_{j}\right)} \end{array}$$

**Figure 4:**
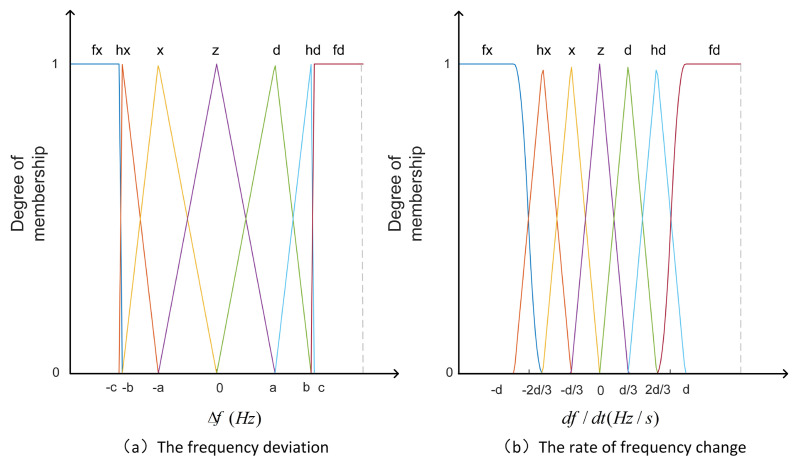
Diagram of fuzzy control input signal membership function.

**Figure 5:**
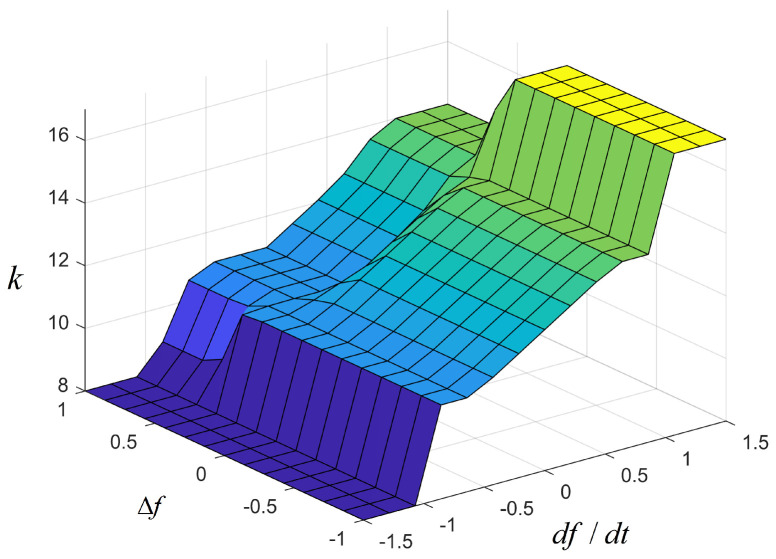
Three-dimensional surface diagram of the fuzzy control rule.

The membership function of the input and output variables is a trigonometric membership function. The trigonometric membership function is used to ensure that the fuzzy output error is zero, which is helpful in adjusting the accuracy of the output active power. The input and output of fuzzy control are divided into seven fuzzy subsets, namely fx, hx, x, z, d, hd, and fd, representing the minimum, smaller, small, zero, large, larger, and maximum, respectively. The range of fuzzy variables taken in this paper are $\Delta f=\left[-1,1\right]$, $df/dt=\left[-1.5,1.5\right]$ and $k=\left[5,20\right]$. Figure 4 is the fuzzy controller input membership function. Figure 5 is the three-dimensional diagrams of the fuzzy inference.

The fuzzy rules are shown below. At medium and low wind speed, *k* increases with the increase in df/dt. When both Δf and df/dt are small, the value of *k* should be small. When Δf is small and df/dt is large, the value of *k* should be small. When Δf is large and df/dt is small, the value of *k* should be larger. When both df and df/dt are large, the value of *k* should be larger. The fuzzy logic reasoning table is shown in Table 1.

## 3. Improved ABC Algorithm to Optimize Fuzzy Control

The core of fuzzy control is the determination of fuzzy membership functions. Since the general membership function is determined by historical experience, a large number of experiments are required to correct it. In this paper, the improved ABC algorithm is proposed to optimize the fuzzy control membership function. The ABC algorithm optimizes the parameters of the fuzzy logic controller. Specifically, the objective function is established to optimize the trigonometric membership function. The ABC algorithm uses a dataset composed of multi-source sensor data for model training and parameter calculation. The multi-source sensor data consist of fluctuating wind speeds and system frequencies. The fuzzy output error can be guaranteed to be zero, and the most suitable primary frequency modulation coefficient can be obtained. Ergodic, irregular, and random chaotic sequences are used to obtain the optimal type instead of the original random sequence. The neighborhood search mechanism is used to move to the better solution while abandoning the poor solution. The ABC algorithm adopts the probabilistic stochastic method when updating new solutions, which improves the global convergence and avoids the property of a local single point.

### 3.1. Improved ABC Algorithm

The objective function is OF. We optimize the membership function to obtain the optimal fuzzy membership function parameters, and then obtain the reference power output of the wind farm and battery. OF is (8).

(8) $$OF\left(a,b,c,d\right)=\zeta OS+\psi \int |\Delta f{|}^{2}dt+\mu t_{s}$$

where ζ, ψ and μ are the weighting coefficients of the objective function.

(9) $$\left\{\begin{array}{c} \zeta =\sqrt{a^{2}+b^{2}+c^{2}}\\ \psi =d/2\\ \mu =0.2(a+b+c) \end{array}\right.$$

where OS is the maximum overshoot observed by the active compensation power and $t_{s}$ is the settling time of the coordinated frequency response.

**Figure 6:**
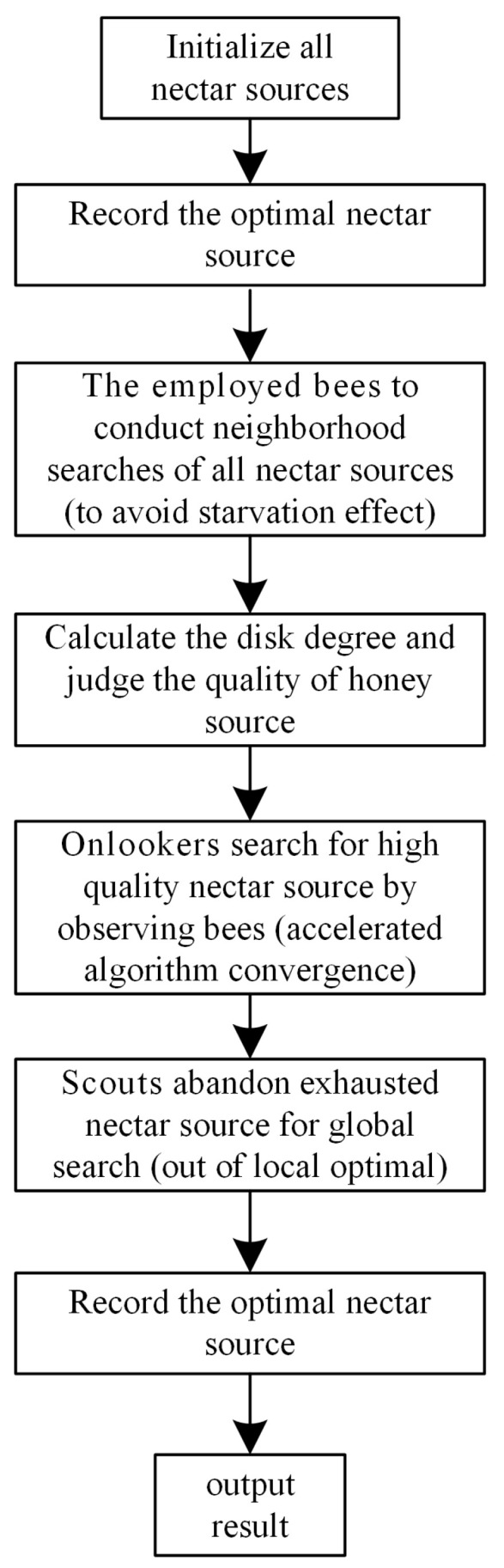
Flow chart of the ABC algorithm.

ABC simulates the intelligent foraging behavior of bee colonies. It is independent of the initial solution and has no derivatives, no trapping in local extrema, and no divergence cases. Bee colonies are divided into three categories: employed bees, onlookers, and scouts. The employed bees and the scouts are equal, each accounting for half of the colony, and the number is equal to the number of nectar sources. Each nectar source can only have one employed bee collecting honey at a time. The roles of scouts and the employed bees can switch with each other. Figure 6 shows the algorithm flow.

(1) The employed bees. Compare the random initial solution created by the ABC algorithm $\left\{x_{1},x_{2},x_{3},\ldots ,x_{Eb}\right\}$ with the newly discovered solution, and preserve the solution with high nectar content, where Eb is the number of employed bees and $x_{i}$ is the D-dimensional vector. D represents the parameters to be optimized.
  Introduce Lévy flight into the ABC to expand the search space and enhance the global search capability. The Lévy distribution is (10).
  (10) $$levy∼u=t^{-\beta },1\leq \beta \leq 3$$
  where *u* and *t* are parameters that obey the normal distribution. β is a number in the range [1, 3].

(2) Onlookers. Select a new solution. Each onlooker bee searches according to the solution of the employed bee. The probability $p_{i}$ of onlookers choosing the food source is calculated as follows:
  (11) $$p_{i}=\frac{fitness_{i}}{\sum_{i=1}^{E_{b}}fitness_{i}}$$
  The search for a new solution can be calculated using (12).
  (12) $$v_{ij}=\left\{\begin{array}{c} x_{ij}+u_{ij}\left(x_{ij}-x_{kj}\right),R_{ij}<MR\\ x_{ij},\;\mathrm{others} \end{array}\right.$$
  where $v_{ij}$ is the new solution after selective processing, while $j\in \left\{1,2,\ldots ,D\right\}$ and k≠i are randomly selected numbers. ($k\in \left\{1,2,\ldots ,E_{b}\right\}$) is the total number of food source locations.The multiplier $u_{ij}$ ($u_{ij}\in \left[-1,1\right]$) is a random number. In other words, $x_{ij}$ is the *j*-th parameter of solution $x_{i}$ which is chosen to be modified.
  By introducing the control parameter modification rate $MR\in \left\{0,1\right\}$, when MR is too large, the improvement effect of the algorithm is not obvious, and the optimization effect of the algorithm is too small. We update the solution when the uniformly distributed random number $R_{ij}$ is less than MR. In other cases, the new solution does not change. The convergence speed of the algorithm is improved by improving the search formula of the solution.
(3) Scouts. If the employed bees and the onlookers do not find a better solution during the search, they become scouts. Each scout randomly selects a new solution in the solution space and updates the current optimal solution. If a new solution generated by a scout bee is better than the current optimal solution, the new solution will be replaced with the current optimal solution. The new solution can be calculated by (13).
  (13) $$x_{ij}=x_{j}^{min}+r\left(x_{j}^{max}-x_{j}^{min}\right)$$
  where *r* is a random number between [−1, 1]; $x_{j}^{max}$ and $x_{j}^{min}$ are the upper and lower bounds of the *j*-th dimension.

The convergence speed of the ABC algorithm to optimize fuzzy control parameters is slow. The artificial ant colony algorithm was optimized by introducing the Gaussian walk mechanism. When a local optimal solution is encountered or stalled during the search process, a new position can be generated by Gaussian walk. This new position is obtained by adding the original position to a random step that follows a Gaussian distribution. In this way, the algorithm can escape the limitation of the local optimal solution and continue to search in the global scope. The ABC algorithm is used to generate perturbations by generating new individuals near the optimal individuals. Thus, local optimal results are avoided and convergence is accelerated. The step size calculation formula is (14). The perturbation formula of the Gaussian walk mechanism is (15).

(14) $$\tau =\frac{\log(i)}{i}$$

(15) $$\begin{array}{c} x\left(i+1\right)=x\left(i\right)+\tau Gaussian(x\left(i\right),\left|x^{best}\left(i\right)\right|)\\ -(r_{1}x^{best}\left(i\right)-r_{2}x\left(i\right)) \end{array}$$

where τ is the step size. *i* is the number of iterations. Gaussian() is the Gaussian distribution function. $x^{best}$ is the optimal individual of the current population. $r_{1}$ and $r_{2}$ are random numbers that obey an even distribution between [0, 1].

The key control parameters of the ABC algorithm are as follows:

(1) Population size $C_{s}$ consists of the number of employed bees $E_{b}$ and $O_{b}$ onlookers;
(2) The limit value, which is the number of trials in which the location of the food source is abandoned;
(3) The maximum number of cycles, MCN.

The improved ABC algorithm contains Lévy flight to carry out a neighborhood search. The problem that the local fitness function value is retained for a long time and falls into local optimal is solved. The ABC algorithm uses Gaussian random walks to perturb all of the individuals in the entire population. It helps individuals to escape the local optimal solution and speeds up the convergence of the algorithm.

### 3.2. Fuzzy Controller Parameter Optimization Based on the ABC Algorithm

**Figure 7:**
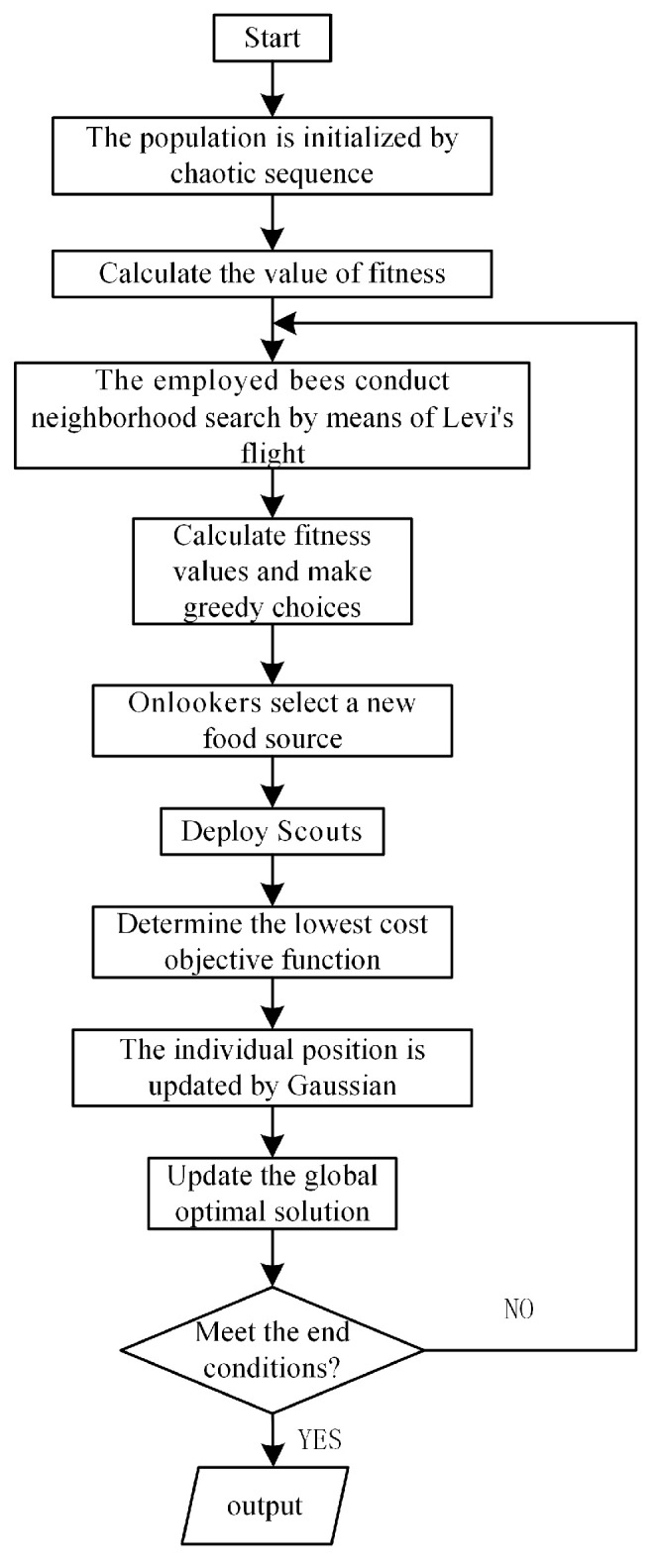
Flowchart of ABC algorithm optimization.

**Figure 8:**
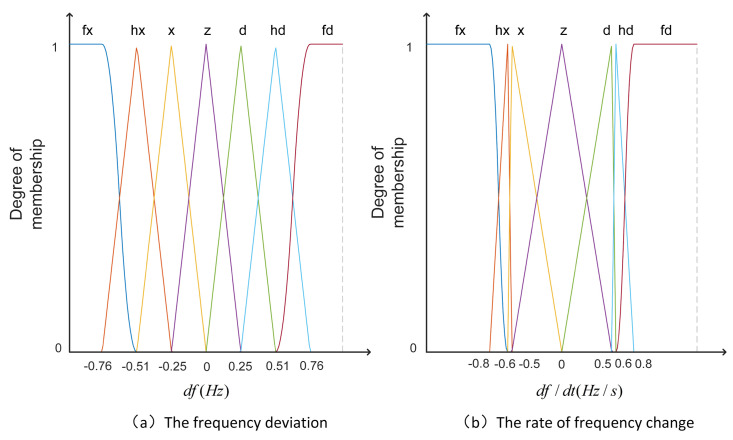
The optimized diagram of fuzzy control input signal membership function.

**Figure 9:**
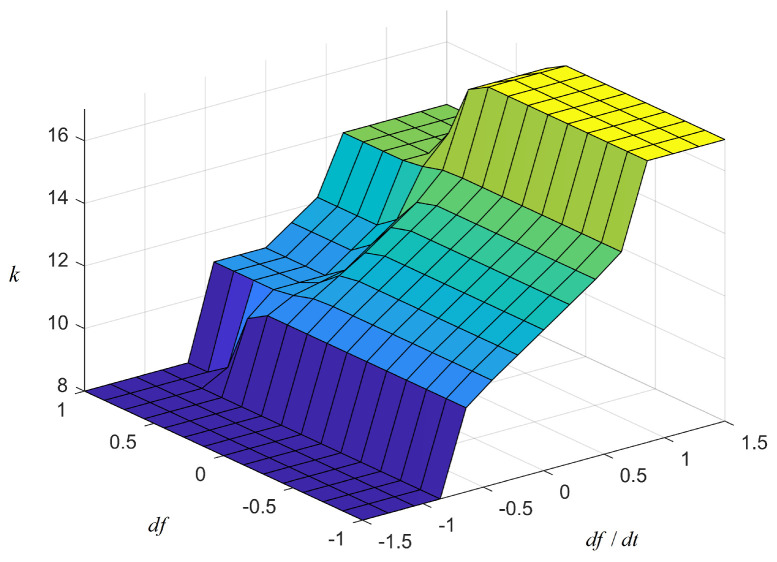
The optimized 3D surface diagram of the fuzzy control rules.

The fuzzy control parameters are optimized by improving the ABC algorithm. The ABC algorithm was set to optimize parameters for 50 iterations, and the number of bee colonies was 10. Figure 7 shows the flowchart. Figure 8 is the fuzzy controller input membership function optimized by the ABC algorithm. Figure 9 is the fuzzy inference 3D graph optimized by the ABC algorithm.

## 4. Simulation and Analysis

**Figure 10:**
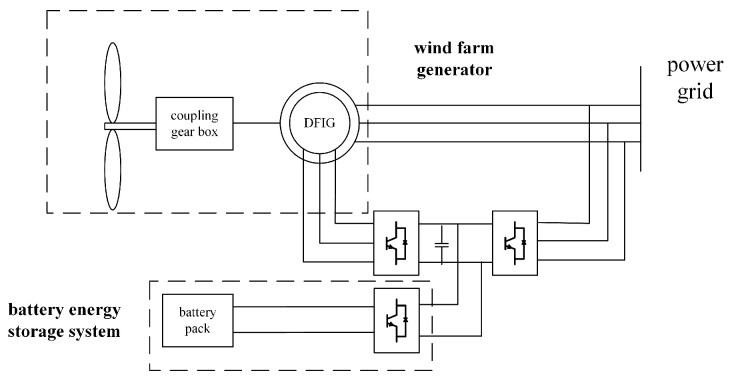
Wind farm and BESS topology.

In the simulation, the wind power penetration rate is set to 25%, with the SOC of 0.8 and wind speeds ranging from 5 m/s to 12 m/s. The load is 100 MW, and the rated power of the wind farm is 200 MW. The rated power of the energy storage system is configured to be 10% of the wind farm’s rated power, with a storage capacity of 50 kWh. The relationship between wind generator power and battery capacity can be seen in related articles [11]. The system suddenly experiences a load of 0.1 p.u. at 2 s. This paper uses the MATLAB R2021b/Simulink simulation platform to construct a combined system consisting of a DFIG and a BESS, as shown in Figure 10. A fuzzy controller was designed using the fuzzy toolbox, and simulation experiments were conducted.

This section verifies the adaptability of the wind farm and BESS coordinated system, optimized by the ABC algorithm based on multi-source data, to load fluctuations. The relevant parameter settings for damping characteristic *D*, rotor Inertia response time constant $T_{\omega }$, blade angle pitch control response time constant $T_{\beta }$, and energy storage response time constant $T_{E}$ are shown in Table 2.

### 4.1. Simulation Analysis of Coordinated System of Wind Farm and BESS at Medium Wind Speed

When the wind speed is at medium level, the coordinated frequency modulation model of a wind farm and a BESS proposed in this paper is simulated and verified. The wind speed is 12 m/s, and the abrupt increase in the load of the system is 0.1 p.u. at 2 s. The frequency modulation control without energy storage and only relying on the wind farm itself [14], the fuzzy control coordinated primary frequency control strategy of the wind farm and the BESS [28], and the improved ABC algorithm proposed in this paper are simulated and compared.

In the frequency modulation control without energy storage and only relying on the wind farm itself [14], the wind farm employs inertial control to regulate the mechanical components of the turbine and ensure stable operation of the generator. Blade angle pitch control is used to maximize the capture efficiency of wind energy. By combining these two methods, the wind turbine system achieves stable and efficient operation under various wind speeds and operating conditions. In the fuzzy control coordinated primary frequency control strategy of the wind farm and the BESS [28], multivariable fuzzy control is applied to dynamically select virtual inertia control coefficients and droop control systems, aiming to enhance the frequency regulation capability of the system.

**Figure 11:**
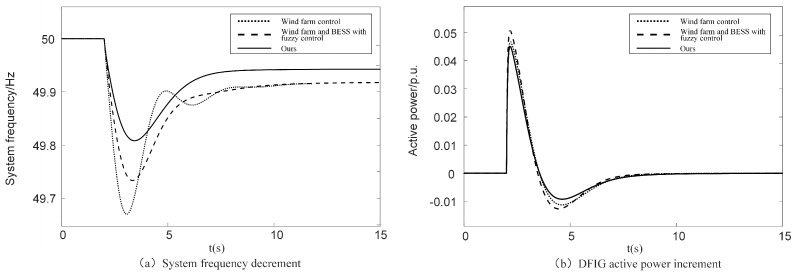
Comparison of simulation results load abrupt increase under different frequency modulation control strategies of medium wind speed.

The simulation results of the abrupt increase in load at a medium wind speed are shown in Figure 11 and Table 3.

As can be seen in Figure 11a, when an abrupt increase in load causes a drop in system frequency, only relying on the wind farm control leads to the largest frequency drop value, which is 49.66 Hz, and the response speed is the slowest. The addition of a fuzzy control increases the minimum frequency by 0.07 Hz. The response speed is greatly improved compared with only relying on the wind farm control. The control optimization strategy proposed in this paper increases the lowest point of frequency drop from 49.73 Hz to 49.81 Hz compared with the unoptimized fuzzy control. This is because the energy storage system provides inertia compensation for the SOC-based DFIG through multi-input fuzzy control. It improves the frequency response characteristics of DFIG.

As can be seen from Figure 11b, when the frequency drops, the active output of the DFIG increases and then decreases, and finally returns to the initial value. This is because when the frequency drops, the DFIG reduces its rotational speed and releases the energy of the rotor movement to participate in frequency modulation. With the continuous decrease in speed, when the frequency modulation capacity coefficient reaches the lower limit value threshold, in order to maintain the safe and stable operation of the DFIG, the DFIG gradually withdraws from the frequency modulation with the decrease in the frequency modulation capacity coefficient. The control strategy proposed in this paper outputs the highest active power, fully utilizing the frequency modulation capability of DFIG, resulting in the fastest frequency response speed and the best primary frequency modulation effect.

**Figure 12:**
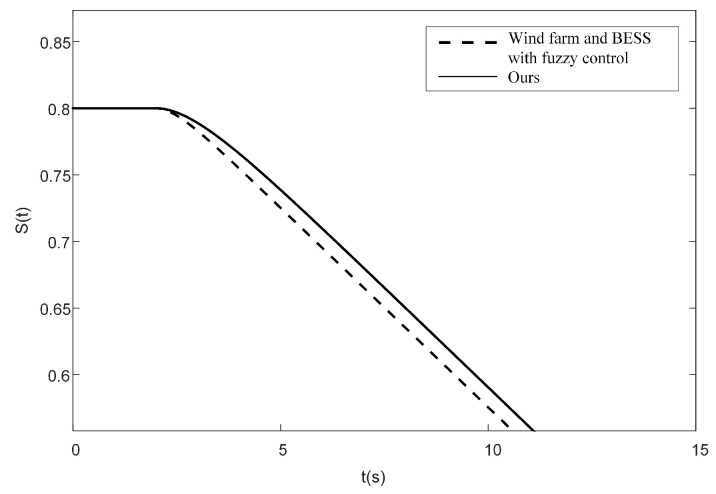
SOC change curves of different control methods.

Under medium wind speed, the load suddenly increases at 2 s, causing the battery capacity to begin changing, as shown in Figure 12. It can be observed that when the energy storage system participates in frequency regulation with an initial SOC of 0.8, the control method proposed in this paper results in a more gradual SOC change curves. This method significantly suppresses fluctuations in the SOC, there by preventing the energy storage system from prematurely exiting frequency regulation due to rapid changes in the charging state.

**Figure 13:**
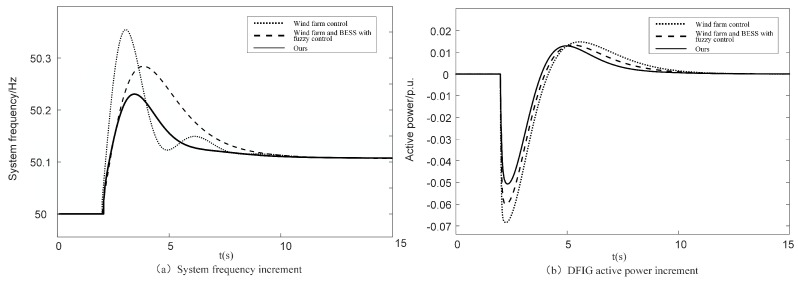
Comparison of simulation results load abrupt decrease under different frequency modulation control strategies of medium wind speed.

Figure 13a shows the frequency curve of the system when the load is abruptly reduced by 0.1 p.u. at 2 s. Table 4 is the specific value of the frequency changes of abrupt decrease in load at mediun wind speed. Among the three control methods, the proposed method takes the shortest time in the frequency modulation process. When wind farm is modulated separately, the frequency rise speed is the fastest and the highest frequency point is the highest. The optimization strategy and fuzzy control proposed in this paper are improved at the highest frequency point. Compared with the state of an abrupt increase in load in the primary frequency modulation, the steady-state frequency deviation has no obvious advantage. The steady-state frequency of all three methods is 51.1 Hz.

### 4.2. Simulation Analysis of Coordinated System of Wind Farm and BESS at Low Wind Speed

**Figure 14:**
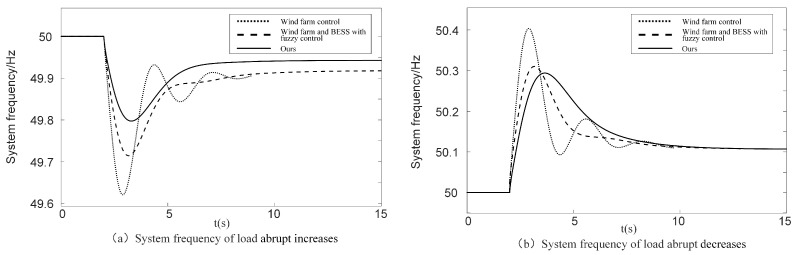
Frequency change curves of abrupt changes in load at low wind speed.

When the wind speed is at a low level, the simulated and verified coordinated frequency modulation model of the wind farm and BESS proposed in this paper is applied. The wind speed is 5 m/s, and the load changes abruptly at 2 s. We simulate and compare the frequency modulation control relying solely on the wind farm without energy storage, the fuzzy control coordinated primary frequency control strategy of the wind farm and BESS, and the ABC algorithm-optimized fuzzy control frequency modulation strategy proposed in this paper. Figure 14 shows the simulation results of the abrupt change in load at a low wind speed, with an abrupt increase in load of 0.1 p.u. in Figure 14a and an abrupt decrease in load of 0.1 p.u. in Figure 14b. Table 5 is the specific value of the frequency changes of abrupt changes in load at low wind speed.

Comparing Figure 11 with Figure 13, it can be seen that, at low wind speeds, the steady-state frequency deviation and maximum frequency deviation of the proposed method are the smallest, regardless of abrupt increases or decreases in load. The steady-state frequency deviation is reduced by 14%. Compared with a low wind speed, the frequency modulation performance of the system at a medium wind speed is better and the frequency recovery time is shorter. The reason for the remarkable effect of a medium wind speed is that the DFIG does not participate in the frequency modulation of the system at low wind speeds. The DFIG rarely participates in the frequency modulation of the system, and mostly relies on the frequency modulation power output of the energy storage system. In the medium-wind-speed area, it can give full play to its frequency modulation ability. Moreover, the frequency modulation situation in the medium-wind-speed area is complex, and the fuzzy control can adjust the system parameters in real time according to the wind speed and frequency deviation. The system achieves a better frequency modulation effect. The simulation results of the continuous load fluctuation experiment at medium wind speed are shown in Appendix A, including DFIG active power, frequency change curves, and other waveforms.

## 5. Discussion

In this paper, a wind farm and a BESS are combined and used to form a coordinated system of a wind farm and BESS. Based on the multi-source sensor data, the ABC algorithm optimizes fuzzy control parameters. An optimal control strategy is proposed for the participation of the coordinated system of a wind farm and a BESS in the primary frequency modulation process of the power grid under multi-source sensor data. This study is of great significance for the stability and resilience of high-permeability wind power generation systems in the future. Through theoretical analysis and simulation verification, the following conclusions are drawn:

(1) The frequency modulation method proposed in this paper is compared with the frequency modulation of the wind farm with the addition of fuzzy control, but the waveform is not optimized. It is concluded that fuzzy control is added, and the ABC algorithm is used to optimize the frequency response speed. This method significantly improves the frequency stability of the system.
(2) In this paper, the Gaussian walk mechanism is introduced into the ABC algorithm to optimize the artificial ant colony algorithm to improve the convergence speed. The ABC algorithm under multi-source sensor data provides great improvement for the performance of the proposed fuzzy controller. The ABC algorithm greatly simplifies the correction steps of the fuzzy logic functions.
(3) This article proposes an intelligent algorithm to optimize the frequency conversion strategy of wind farms and BESSs based on multi-source data consisting of wind speed and frequency. This aims to provide more accurate and reliable frequency regulation services for integrating renewable energy into the grid. It contributes to the automation and informatization of microgrids. With the development of monitoring and transmission equipment such as sensors, frequency regulation control will become more precise and rapid. Moreover, it enhances the intelligence and operational efficiency of the distribution network, ensuring the stability and continuity of the power supply.

## Figures and Tables

**Table 1:** Fuzzy rules of fuzzy reason.

df/dt				Δf			
	fx	hx	x	z	d	hd	fd
fd	hx	hx	hx	hx	hx	hx	hx
hx	x	x	x	x	x	x	hx
x	x	x	x	x	x	x	hx
z	z	z	z	z	z	x	x
d	d	d	d	d	d	z	z
hd	hd	hd	hd	hd	hd	d	d
fd	fd	fd	fd	fd	fd	hd	hd

**Table 2:** Parameter associated with coordinated primary frequency control system of wind farm and BESS.

Parameter	Parameter Symbol	Value
Damping characteristic	*D*	2
Rotor inertia response time constant	$T_{\omega }$	0.1
Blade angle pitch control response time constant	$T_{\beta }$	3
Energy storage response time constant	$T_{E}$	0.3
system version	/	Window11
CPU	/	Intel(R) Core(TM) i7-1065G7
GPU	/	NVDIA GeForce RTX 3080

**Table 3:** Comparative analysis of the frequency of an abrupt increase in load at medium wind speed.

Model of Frequency Control	The Lowest Frequency	Steady-State Frequency Deviation	Maximum Frequency Deviation
Ref. [14]	49.66	0.09	0.34
Ref. [28]	49.73	0.09	0.27
**Ours**	**49.81**	**0.07**	**0.19**

Note: Unit: Hz. The bold text is the experimental data of this paper.

**Table 4:** Comparative analysis of the frequency of load abrupt decrease at medium wind speed.

Model of Frequency Control	The Highest Frequency	Steady-State Frequency Deviation	Maximum Frequency Deviation
Ref. [14]	50.35	0.09	0.35
Ref. [28]	50.28	0.09	0.28
**Ours**	**50.23**	**0.09**	**0.23**

Note: Unit: Hz. The bold text is the experimental data of this paper.

**Table 5:** Comparative analysis of the frequency changes of abrupt changes in load at low wind speed.

	Model of Frequency Control	Frequency Extreme Points	Steady-State Frequency Deviation	Maximum Frequency Deviation
Abrupt increases	Ref. [14]	49.62	0.08	0.38
	Ref. [28]	49.71	0.08	0.29
	**Ours**	**49.80**	**0.06**	**0.20**
Abrupt decreases	Ref. [14]	50.41	0.12	0.41
	Ref. [28]	50.31	0.12	0.31
	**Ours**	**50.28**	**0.12**	**0.28**

Note: Unit: Hz. The bold text is the experimental data of this paper.

**Figure A1:**
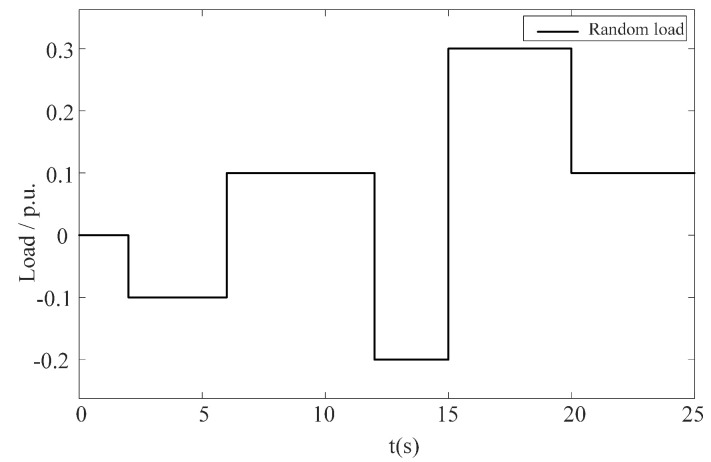
Continuous load variation.

**Figure A2:**
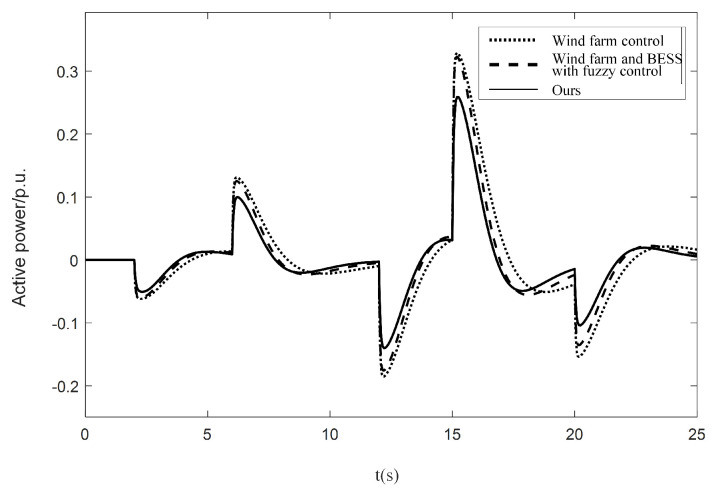
DFIG active power change curve.

**Figure A3:**
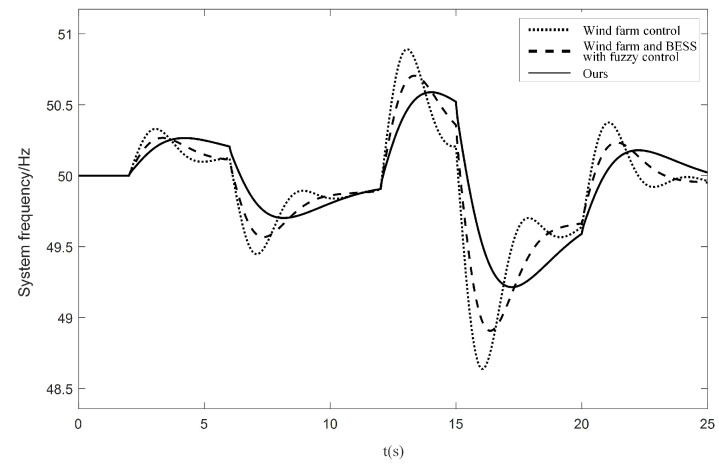
Frequency change curves.

## Data Availability

Data is contained within the article.

## Appendix A

In the text, the effectiveness of the primary frequency modulation control strategy of the wind storage system is verified when the load only changes once. The steady-state frequency deviation and the maximum frequency deviation are the smallest, and the steady-state frequency deviation is reduced by 14%. In order to verify whether the control strategy proposed in this paper can cope with continuous irregular load fluctuations, the operation state of continuous load fluctuations is simulated. The load change conditions are as follows: an abrupt decrease in the load of the system of 0.1 p.u. at 2 s, an abrupt increase in the load of the system if 0.2 p.u. at 6 s, an abrupt decrease in the load of the system of 0.3 p.u. at 12 s, and abrupt increase in the load of the system of 0.5 p.u. at 15 s, and an abrupt decrease in the load of the system of 0.2 p.u. at 20 s. The system output power also fluctuates with the load fluctuation. The output power decreases during sudden load reductions and increases during sudden load increases. When the second load increases sharply, the output power increase is also the largest. Under the adjustment of the system control strategy, the system frequency also fluctuates with the fluctuation of the load, but the change range does not exceed 0.8% of the stable state. The simulation results are shown in Figure A1, Figure A2 and Figure A3.

In a complex power system with random fluctuations in wind speed and load, the proposed method can significantly optimize the frequency stability and response performance of the system. The system frequency deviation and frequency change rate are effectively controlled.

When wind speed fluctuations lead to instability of wind power generation, the DFIG can respond quickly and compensate for or balance this instability by adjusting its active power output. At the same time, the energy storage system acts as a flexible energy buffer pool, which is able to release or absorb energy when needed to further smooth the frequency fluctuations of the system. This synergistic effect not only slows down the frequency change rate, slowing down the large fluctuations in the frequency in a short period of time, but also significantly improves the highest and lowest points of the system frequency, reducing the amplitude of the frequency offset, making the system frequency closer to the rated value, and improving the stability and reliability of the power system.
